# Protected high risk percutaneous coronary intervention—Impella 5.0 as a single-access technique: a case report

**DOI:** 10.1093/ehjcr/ytae060

**Published:** 2024-01-30

**Authors:** Marta Medina, Philip Wenzel, Bilel Fathallah, Tobias Ruf, Mehmet Oezkur

**Affiliations:** Department of Cardiac Surgery, University Medical Center of the Johannes Gutenberg University Mainz, Langenbeckstraße 1, Mainz 55131, Germany; Department of Cardiology, University Medical Center of the Johannes Gutenberg University Mainz, Mainz, Germany; Department of Cardiac Surgery, University Medical Center of the Johannes Gutenberg University Mainz, Langenbeckstraße 1, Mainz 55131, Germany; Department of Cardiology, University Medical Center of the Johannes Gutenberg University Mainz, Mainz, Germany; Department of Cardiac Surgery, University Medical Center of the Johannes Gutenberg University Mainz, Langenbeckstraße 1, Mainz 55131, Germany

**Keywords:** Percutaneous microaxial flow pump, Protected high-risk PCI, Impella 5.0, Single-access technique, Angio-seal technique, Case report

## Abstract

**Background:**

Patients requiring coronary intervention after acute myocardial infarction, with decompensated heart failure and multiple co-morbidities, present a challenging clinical scenario. Addressing such high-risk cases has been a marked increase in the simultaneous support using microaxial flow pump devices, providing a crucial haemodynamic support during procedures.

**Case summary:**

We report the case of a 58-year-old man, with a non-ST-segment elevation myocardial infarction in the context of a peripheral vascular surgery. Echocardiography revealed severely reduced left ventricular function and cardiac magnetic resonance imaging demonstrated transmural scars in all but left anterior descending artery area. The patient was of extreme high surgical risk due to the multiple co-morbidities, acute decompensation heart failure, and peripheral artery disease, and, therefore, the heart team preferred protected percutaneous coronary intervention (PCI) over coronary artery bypass graft for revascularization. The peripheral artery disease included severely calcified ascending aorta, occlusions of both femoral arteries, the left subclavian artery, and the right radial artery. Taken together, the heart team agreed on a hybrid approach with surgical implantation of Impella 5.0 via the left subclavian artery, by a single-access technique. Following the intervention procedure, haemostasis of the vascular prosthesis was achieved by an angio-seal technique without complications. The patient recovered satisfactorily, with improved left ventricular function, and discharged 10 days post-procedure.

**Discussion:**

The single-access high-risk PCI technique offers a standardized approach for microaxial flow pump devices such as Impella 5.0 and PCI. The subclavian artery as a single-access route for high-risk PCI has demonstrated safety and efficacy.

**Learning points**

- To describe the feasibility of a single-access approach for Impella support in patients requiring high-risk percutaneous coronary intervention (HR-PCI) with severe peripheral arterial disease.
- To be able to use the angio-seal technique as a safe technique for haemostasis of the vascular prosthesis.
- To understand the crucial role of employing a temporary microaxial flow pump in appropriately selected patients undergoing HR-PCI.

## Introduction

Patients requiring coronary intervention following an acute myocardial infarction frequently develop acute decompensated heart failure, evidenced by poor left ventricular function and co-morbidities that consequently amplify the associated risks of intervention. This specific patient population raises an ongoing challenge marked by high in-hospital mortality and unfavourable outcomes.^1^ Over the last decade, there has been a significant increase in the number of patients, characterized by a higher load of co-morbidities and more complex coronary artery disease, who are being considered for technically challenging and high-risk percutaneous coronary interventions (HR-PCI). Simultaneously, the development of a new generation of mechanical circulatory assist devices, such as the microaxial flow pump (mAFP), which offers improved haemodynamic support and reduced device-associated complications, combined with strategic timing of device utilization in the context of HR-PCI, has played a pivotal role in providing crucial haemodynamic support during the intervention.^2^ These procedures performed with the support of mAFP aim not only to provide haemodynamic support but also to unload the left ventricle, allowing for safe and successful coronary revascularization.

## Summary figure

Suggested algorithm for protected HR-PCI supported by Impella 5.0 and using the right subclavian artery as the single access technique.

**Figure:**
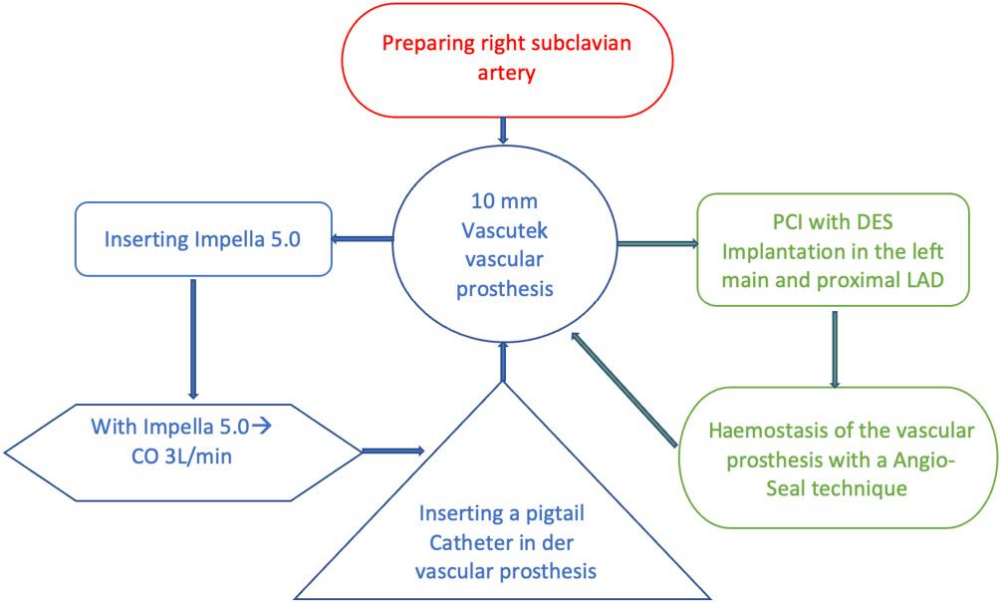


## Case presentation

A 58-year-old man with terminal heart failure was admitted to our cardiology department with a non-ST-segment elevation myocardial infarction (NSTEMI) and acute cardiac decompensation following vascular surgery.

The patient had a clinical history of acute coronary syndrome and resuscitation. After the mentioned surgical intervention, the patient developed dyspnoea and respiratory insufficiency, suggesting cardiac decompensation. The patient was normotensive without the use of catecholamines, and an electrocardiogram revealed a pre-existing left bundle branch block. In addition, there was an elevated troponin level of 2660 pg/mL (limit > 15 pg/mL), suggesting the possibility of an acute coronary syndrome. An emergency cardiac catheterization revealed high-grade stenosis in the main trunk and proximal left anterior descending (LAD) artery, along with occlusion of the right coronary artery with retrograde perfusion. Transthoracic echocardiography revealed severely impaired left ventricular function [left ventricular ejection fraction (LVEF) = 12%], with posterior wall akinesia and residual diffuse hypokinesia (see Supplementary material online, *Video S1*). Magnetic resonance imaging showed a transmural scar in the posterior and lateral walls, as well as a partially vital myocardium in the LAD area.

**Figure 1:**
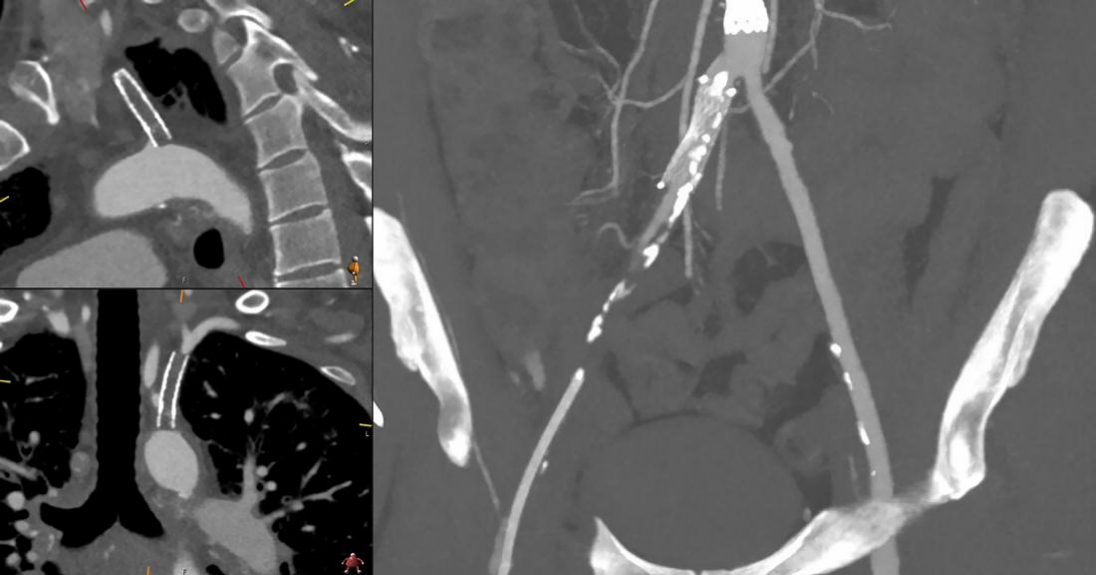
Computed tomography angiography of proximal arteries with peripheral artery disease. Images of the occluded left subclavian artery, both femoral arteries and the left radial artery.

Peripheral artery disease (PAD) was also observed (*Figure 1*), including a severely calcified aorta and occlusion of the left subclavian artery, left radial artery, and both femoral arteries. The severely calcified ascending aorta prevented on-pump surgery and the option for a central anastomosis. The occluded left subclavian artery did not allow a minimally invasive direct coronary artery bypass grafting (CABG) procedure, and the history of advanced chronic obstructive pulmonary disease, obstructive sleep apnoea, type 2 diabetes mellitus, and advanced nicotine dependence classified the risk of surgical revascularization to be high. Therefore, a protected HR-PCI using a mAFP device (Impella 5.0) and the right subclavian artery as a single-access route was chosen.

### Management

Under general anaesthesia and upon exposing the right subclavian artery and achieving an activated clotting time of more than 250 s via administration of unfractionated heparin, a 10 mm Vascutek vascular prosthesis was put into place. The Impella 5.0® system (Abiomed, Danvers, MA, USA) was positioned in the left ventricle under transoesophageal echocardiography control. The flow was set and maintained at 3 L/min at level p3 without complications.

**Figure 2:**
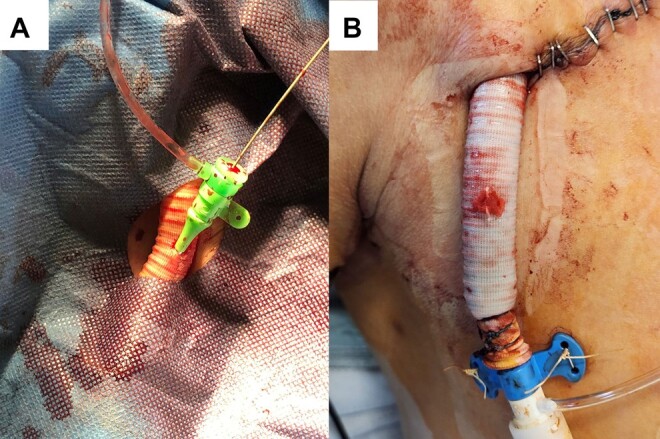
Pigtail catheter for percutaneous coronary intervention procedure and haemostasis closure with angio-seal technique. (*A*) Pigtail catheter via soft Terumo 6 Fr Glidesheath Slender in the medial third of the vascular prosthesis to perform the percutaneous coronary intervention. (*B*) Haemostasis of the vascular prosthesis using an angio-seal technique.

**Figure 3:**
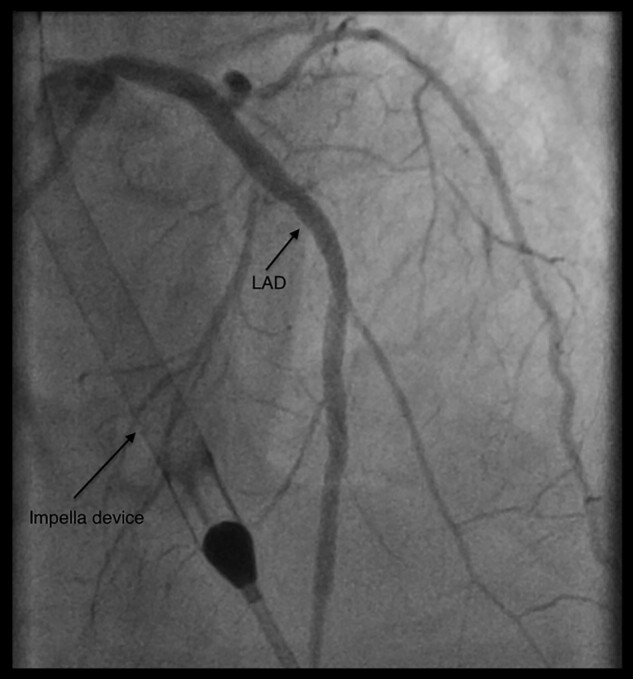
Final angiography showing good outflow tract through the coronary arteries following drug-eluting stent insertion in the left main and proximal left anterior descending coronary. LAD, left anterior descending.

After achieving a constant cardiac output of 3 L/min with the Impella, we proceeded to perform the PCI by inserting a pigtail catheter via soft Terumo 6 Fr Glidesheath Slender in the medial third of the vascular prosthesis (*Figure 2A*). In a Xience Pro X (3.5 × 48 and 3.0 × 48 mm), drug-eluting stents were deployed in the left main and proximal LAD coronary (see Supplementary material online, *Video S2*). The subsequent control angiography showed a good primary result, without evidence of residual stenosis or dissection, and with free flow far into the periphery (*Figure 3*). Following this procedure, we achieved haemostasis of the vascular prosthesis using an angio-seal technique. A 6 Fr angio-seal vascular closure device (ANGIO-SEAL VIP Vascular Closure Device, Terumo International Systems, Somerset, NJ, USA) was deployed over the wire (see Supplementary material online, *Video S3*). This device creates a mechanical seal by sandwiching the vascular prosthesis between a stable bioabsorbable anchor and collagen sponge, without risk of embolism (*Figure 2B*).

The patient was stabilized post-procedure and the weaning of the Impella 5.0 was initiated.

### Follow-up

After the successful removal of the device 6 h later, the patient experienced an excellent recovery, with an improved LVEF of 30% and was discharged 10 days later.

## Discussion

Coronary artery bypass grafting and PCI have been extensively studied in patients with coronary artery disease and poor LVEF.^3^ In the current management of NSTEMI, the timing of PCI is dependent on risk stratification because the benefit of an early invasive strategy is evident in high-risk patients. The early transfer and invasive intervention of high-risk patients with NSTEMI may impact the cardiac survival benefit.^4^ Microaxial flow pump device is often required in these cases to increase cardiac output and mean arterial pressure while minimizing the impact on coronary blood flow, providing adequate haemodynamic support during the HR-PCI.^5^ This implies that initiating haemodynamic support early with a mAFP before HR-PCI prevents the subsequent cascade of ischemia and hypoperfusion,^6^ with a favourable impact on short- and medium-term mortality compared with employing mAFP support after HR-PCI.^7^

In this case, considering the concomitant PAD, which is a frequently concomitant disease in this type of patients, a single-access technique for HR-PCI becomes relevant. The single-access HR-PCI technique has been described,^8^ providing a standardized approach for mAFP such as Impella CP and HR-PCI.^9,10^ To ensure a safe procedure, previous computed tomography scans should be reviewed to assess the calcification and tortuosity of the arterial system. The right subclavian artery was the only viable access route in our patient; therefore, we modified the single-access technique using the Impella 5.0 access vascular prosthesis for HR-PCI, providing a safe and feasible access route allowing full systemic flow and left ventricular loading. Following the intervention, it was crucial to achieve sufficient haemostasis of the vascular prosthesis. Therefore, the use of the angio-seal technique offers to be not only a rapid but also a safe method, ensuring that sufficient haemostasis is achieved.

## Conclusion

The subclavian artery has demonstrated its safety and efficacy as a single-access route for HR-PCI. However, due to the potential complications associated with this procedure, a thorough evaluation of vascular access using computed tomography angiography is crucial.

## Supplementary Material

ytae060_Supplementary_Data

## Data Availability

The data underlying this article are available in the article and in its online supplementary materials.

## Lead author biography

Marta Medina, MD, who is affiliated in the Department of Cardiac and Vascular Surgery University Medical Center of the Johannes Gutenberg University Mainz, Germany, completed her medical studies at the University Pontificia Javeriana, Bogota Colombia. She began her residency in cardiac surgery in Germany, at the University Hospital RTWH Aachen, then at University Hospital Bonn, and currently at the University Medical Center of the Johannes Gutenberg University Mainz. Her areas of interest include heart failure, CABG surgery off-pump, and mechanical circulatory support. Dr Medina is an active member of the DGTHG—German Society for Thoracic and Cardiovascular Surgery.
